# Fetal and neonatal samples of a precursor surfactant protein B inversely related to gestational age

**DOI:** 10.1186/1471-2431-13-164

**Published:** 2013-10-10

**Authors:** Christoph Czernik, Gerd Schmalisch, Christoph Bührer, Hans Proquitté

**Affiliations:** 1 Department of Neonatology, Charité University Medical Center, Berlin, Germany; 2 Department of Neonatology / Ped. Intensive Care, University Hospital, Jena, Germany

**Keywords:** C-proSP-B, Blood, Amniotic fluid, Urine, Immaturity, Preterm infants

## Abstract

**Background:**

Alveolar–capillary membrane leaks can increase the amount of surfactant protein B (SP-B) in the bloodstream. The purpose of this study was to measure the concentration of C-proSP-B, a SP-B precursor that includes C-terminal domains, in various body fluids of newborn infants and determine its dependence on gestational age.

**Methods:**

C-pro-SPB was measured in amniotic fluid and umbilical cord blood at birth, and in peripheral blood and urine on postnatal day 3 in 137 newborn infants with a median birth weight of 2015 g (range, 550–4475 g) and gestational age of 34 weeks (range, 23–42 weeks).

**Results:**

C-proSP-B levels differed more than 100-fold among samples. The levels (median; interquartile range) were highest in peripheral blood (655.6 ng/mL; 419.0-1467.0 ng/mL) and lowest in urine (3.08 ng/mL; 2.96-3.35 ng/mL). C-proSP-B levels in amniotic fluid (314.9 ng/mL; 192.7–603.6 ng/mL) were approximately half of those in peripheral blood. In cord blood C-proSP-B was slightly lower (589.1 ng/mL; 181.2-1129.0 ng/mL) compared with peripheral blood. C-proSP-B levels significantly increased in all the fluids sampled except urine with decreasing gestational age (p < 0.001).

**Conclusions:**

This novel assay allows for the quantitative measurement of C-proSP-B in blood and amniotic fluid. The dependence of C-proSP-B on gestational age may hamper its use for the detection of alveolar leaks in preterm newborns.

## Background

Pulmonary surfactant is synthesized by type II pneumocytes and secreted into the airways of the lung beginning at 24 weeks gestation. Its deficiency or dysfunction is the hallmark of respiratory distress syndrome in preterm infants [1,2]. Pulmonary surfactant consists of a complex of lipids and specific proteins, including surfactant proteins (SP) A, B, C and D. Of these surfactant proteins, SP-B is the only one that is absolutely essential for initiating breathing and life [3-5]. The highly hydrophobic SP-B enhances the absorption of phospholipids onto the air–water interface and optimizes surface tension reduction [6,7]. SP-B is a relatively small (79 amino acids) hydrophobic polypeptide that is synthesized as a longer, 381-amino-acid precursor that undergoes a complex maturation process along the exocytic pathway of type II pneumocytes [8]. Processing of the SP-B precursor includes proteolytic cleavage of N- and C-terminal domains in at least three different steps. Cleavage of the N-terminal (24–200) and C-terminal (280–381) flanking domains generates the biophysically active, mature SP-B. C-ProSP-B is a transient intermediate in the synthesis of SP-B that does not accumulate in type II pneumocytes [3].

Although surfactant proteins are normally only found in appreciable amounts in the lung, leakage of SP-A, SP-B and SP-D into the circulation has been reported in a number of respiratory disorders [9-14] and cardiac failure [15-18]. The route by which these proteins enter the circulation is unknown; there is, however, strong evidence that a bidirectional plasma protein flux occurs in the lungs, the magnitude of which depends on disease severity [19,20]. Jobe et al. [21] showed that this bidirectional flux from the alveoli increased as gestational age decreased in prematurely delivered, ventilated lambs.

Currently, there are only a few reports describing the measurement of SP-A levels in cord blood and sera from neonates within 24 hours after birth [22,23]. To date, there are no data regarding C-proSP-B concentrations in the blood of newborn infants. We hypothesized that C-proSP-B could be measured in different samples from newborn infants and that these levels would be correlated with lung maturation. The aim of this exploratory study was to measure C-proSP-B in cord and peripheral blood, amniotic fluid, and urine of preterm and term newborn infants with gestational ages between 24 and 42 weeks.

## Methods

### Patients and protocol

Newborn infants delivered at the Charité University Hospital between October 2009 and May 2011 were enrolled in the study. Infants who died in the first days of life were excluded. The study protocol was approved by the local institutional review board (Ethikkommission der Charité, #EA2/083/09). Written informed consent for sampling was obtained for infants of imminent premature birth before delivery, or on the first day of life in some cases.

### Measurement of C-proSP-B in different samples

Samples of amniotic fluid (5 ml) were collected during labor prior to delivery and umbilical cord blood (1 ml) was obtained at birth. Peripheral blood samples were taken within 36 to 72 hours of age through umbilical artery catheter aspiration or venous puncture as part of routine sampling. Urine was collected in plastic bags at the same time. The samples were centrifuged at 7000 rpm (Eppendorf microcentrifuge) for 3 minutes. Cell-free plasma, urine and amniotic fluid were stored at -20°C until C-pro-SPB analysis. The samples were batched and run together after collection was complete. The tests were performed in a single run and in a blinded manner using a new C-proSP-B electrochemiluminescence immunoassay kit employing a Tris(bipyridyl)-ruthenium(II) complex as a label (Roche Diagnostics, Mannheim Germany), as described by the manufacturer. Briefly, biotinylated capture antibody, ruthenium-labeled detection antibody, and sample or standard material (10 μl) were first incubated in a homogeneous phase for 9 minutes at 37°C. Then, streptavidin-coated beads were added and incubated for 9 minutes to allow binding of the formed immune complexes to the microparticles. After the second incubation, the reaction mixture was transferred to the measuring cell, where beads were captured on the electrode surface by a magnet. The measuring cell was washed to remove unbound label and filled with detection buffer containing Tris-propylamine. After applying voltage to the electrode, the emitted chemiluminescence was detected by a photomultiplier. Results were determined using a two-point calibration curve. C-proSP-B values are given as nanograms per milliliter, and were measurable within a range of 0.1 to 3000 ng/mL.

### Statistical analysis

Patient characteristics were described by median and range and compared using the Mann–Whitney rank test. Measured C-ProSP-B values were described by median and interquartile range (IQR) and compared by using Wilcoxon test for paired samples or the Mann–Whitney rank test for independent samples, as appropriate. Spearman rank correlation coefficients (r_S_) were calculated to investigate the relationship of the C-ProSP-B concentrations between paired samples. A non-linear regression analysis was performed by linearization of nonlinear models to investigate the relationship between C-ProSP-B and gestational age using the Pearson correlation coefficient as a measure of the goodness of fit. Statistical analyses were performed using Statgraphics Centurion® software (Version 16.0, Statpoint Inc., Herndon, Virginia, USA) and MEDCALC (Version 12.2.1.0; MedCalc Software, Mariakerke, Belgium). A p-value < 0.05 was considered statistically significant.

## Results

### Patients

C-proSP-B was measured in 137 patients with a median birth weight of 2240 g (range, 550–4475 g) and a gestational age of 34 weeks (range, 23–42 weeks). 80 (58%) infants were born before 37 gestational weeks (preterm infants) and 17 (12%) infants with a mean gestational age of 28 (24–32) weeks received antenatal steroids. Postnatal mechanical ventilation was necessary for 8 (6%) infants.

A total of 265 C-proSP-B measurements were performed on four different samples; however, not all samples could be obtained from every patient. As shown in Table 1, the largest numbers of measurements were obtained from amniotic fluid (N = 79) and urine (N = 77), whereas the number of blood measurements was lower (peripheral blood, N = 63; cord blood, N = 46).

**Table 1:** C-proSP-B in different samples at which the highest levels were found in the peripheral blood (presented as median and IQR)

**Sample**	**N**	**C-proSP-B (ng/mL)**
**Peripheral blood**	63	655.6 (419.0–1467.0)
**Cord blood**	46	589.1 (181.2–1129.0)
**Amniotic fluid**	79	314.9 (192.7–603.6)
**Urine**	77	3.08 (2.96–3.35)

N = number of samples.

### Comparisons of different samples

**Figure 1:**
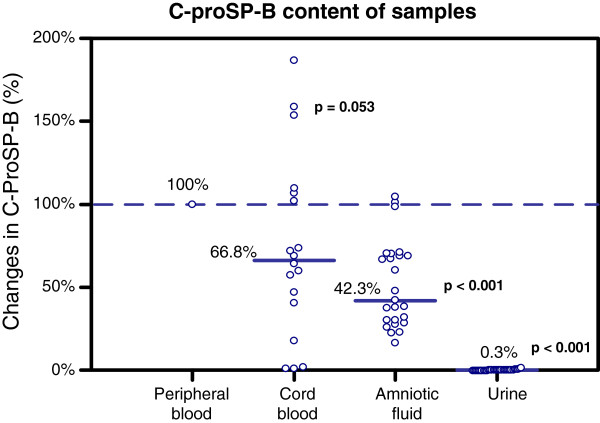
**Paired measurements of the level of C-proSP-B in cord blood, amniotic fluid and urine relative to that in peripheral blood which was set at 100%.** The p-value describes the statistical significance of the differences. (Number of samples see text).

To investigate differences in C-proSP-B levels among the four different samples, we evaluated paired measurements from the same patient (Figure 1). The highest concentrations were detected in peripheral blood, which was used as a reference (defined as 100%); the levels in all other samples were significantly lower. There was a strong correlation between C-proSP-B levels in peripheral and cord blood (N = 18 paired samples, r_S_ = 0.759, p < 0.001). Although C-proSP-B levels in cord blood trended about one-third lower than those in peripheral blood, this difference fell just short of statistical significance (p = 0.053) due to high intersubject variability, which ranged from near 0% to 200% relative to peripheral blood. C-proSP-B levels measured in amniotic fluid were also significantly correlated with C-proSP-B levels measured in peripheral blood (N = 25 paired samples, r_S_ = 0.709, p < 0.001), although they were approximately half those of peripheral blood. The lowest C-ProSP-B concentrations were seen in urine, where levels were only 0.3% of those in peripheral blood; there was only a weak correlation between C-proSP-B levels in these two samples (N = 32 paired samples, r_S_ = 0.483, p = 0.007).

### Dependence of C-proSP-B on gestational age

**Figure 2:**
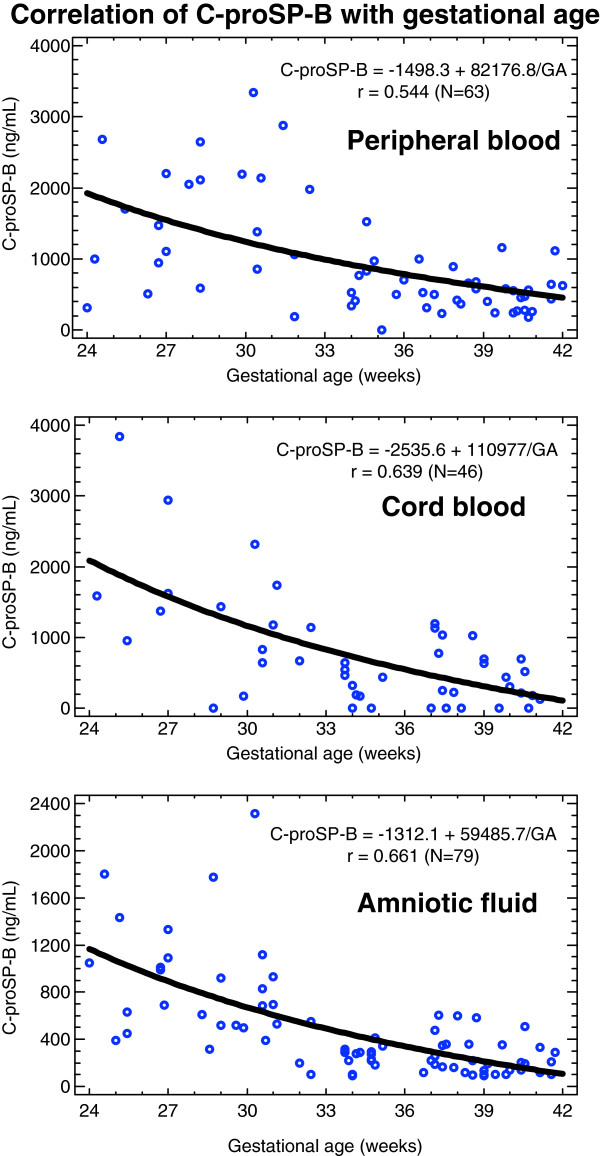
**Increase in C-proSP-B with decreasing gestational age in peripheral (top) and cord blood (middle) and amniotic fluid (bottom).** Presented are the regression curve, the correlation coefficient r and the number of samples N.

With the exception of urine, all samples showed a significant dependence of C-proSP-B levels on gestational age. For each of the remaining three samples, C-proSP-B levels increased significantly (p < 0.001) with decreasing gestational age (Figure 2). In each case, the best correlation was found with the reciprocal value of the gestational age.

The effect of antenatal steroid treatment and mechanical ventilation on C-proSP-B could not be analyzed in this study due to drop outs in the different samples. The incidence of infants who received antenatal steroid treatment and mechanical ventilation in the four sample groups was very small (≤6 and ≤4 infants, respectively).

## Discussion

The study showed that C-proSP-B can be measured in samples of the peripheral and cord blood, amniotic fluid and urine of newborn infants. However, there were distinct differences among the samples investigated. C-proSP-B concentrations differed more than 100-fold among the samples, and were generally highest in the peripheral blood and lowest in urine. C-proSP-B concentrations in peripheral blood were significantly correlated with those in samples of cord blood and amniotic fluid, but only weakly with those in urine samples. Furthermore, with the exception of urine, C-proSP-B levels increased with decreasing gestational age. How C-proSP-B patterns change with further postnatal development is still unknown.

The dependence of C-proSP-B on maturity may hamper its use in preterm infants as an indicator of capillary–alveolar membrane damage caused, for example, by ventilator-induced lung injury, as shown in adults [24,25]. Thus, increased levels of C-proSP-B are difficult to interpret. Although non-invasive methods are currently favored [26,27], intubation and mechanical ventilation are often unavoidable, and the immature lung tissue of these infants is at increased risk of damage. In a review, Takahashi et al. [25] reported that increased levels of surfactant proteins in body fluids reflect alterations of capillary–alveolar membranes and lung maturity. Determining surfactant proteins in body fluids may aid in monitoring the course of pulmonary diseases, such as pulmonary alveolar proteinosis, respiratory distress syndrome and pneumonia. Awasthi et al. [28] showed that in premature newborn baboons, the amounts of SP-A and SP-D in lavage fluid were indicators of infection risk in the pathogenesis of neonatal chronic lung disease.

Moreover, Beresford and Shaw described how lower bronchoalveolar lavage SP-B and SP-D concentrations in preterm infants ventilated for respiratory distress syndrome were associated with worse clinical prognoses [29]. In this context, Keller et al. [30] demonstrated recently in preterm infants that late administration of a surfactant containing SP-B surfactant transiently increases SP-B content in the lung aspirates, possibly leading to improved short- and long-term respiratory outcomes. The diagnostic value of C-proSP-B in newborns in this context is still unknown and will require additional clinical studies. However investigation into the diagnostic value of C-proSP-B as a biomarker should consider that C-proSP-B is highly dependent on gestational age as shown in the present study.

To the best of our knowledge, this is the first study in which C-proSP-B was measured quantitatively in different samples from newborn infants. The four samples investigated are commonly collected for perinatal diagnostics, though it was not possible to obtain all four from every patient. Amniotic fluid can only be obtained non-invasively during delivery. Cord blood is commonly available, though it is difficult to obtain in some patients due to constriction of the vessels. Peripheral blood and urine samples can be collected more reliably, though this requires puncturing a peripheral vessel or using a urine bag, respectively.

It was not surprising that the highest levels of C-proSP-B were found in the peripheral blood. SP-B is a lung-specific protein, so SP-B in amniotic fluid is believed to come directly from the alveolar space, whereas SP-B in blood is thought to have permeated into the circulation from the alveolar space. In this context, SP-B in amniotic fluid transported by fetal breathing movements [31,32] should directly reflect that in the alveolar space, whereas SP-B in blood is influenced by lung permeability, which increases in direct relationship to the severity of lung damage. The lower concentration of C-proSP-B in amniotic fluid than in peripheral blood may be caused by dilution processes, because the amniotic fluid volume is much greater than the infant’s blood volume. The extremely low concentration measured in urine may reflect impaired transport from blood to urine. The renal clearance of proteins is limited by their molecular mass, hydrophobicity and kidney function, which is related to maturity [33].

This pilot study has several limitations. Firstly, as discussed above, we could not obtain peripheral and cord blood, amniotic fluid and urine samples from every patient, hampering the comparability of pro-protein levels among different samples. Secondly, ethical considerations prevented us from performing longitudinal measurements; accordingly, we could not assess the pattern of postnatal C-proSP-B development. Thirdly, due to the low number of patients treated und the sample drop out rate, we could not investigate the effect of prenatal steroid treatment and mechanical ventilation on C-proSP-B. Furthermore, we have no C-proSP-B measurements from infants after long-term ventilator support, meaning the effect of lung injury on C-proSP-B could not be quantified. This would need to be addressed in future clinical studies.

## Conclusions

C-proSP-B can be measured quantitatively in different samples from newborn infants. The highest concentrations of C-proSP-B were found in peripheral blood. With the exception of urine, C-proSP-B levels increased with decreasing gestational age, a factor that should be considered in the interpretation of elevated levels.

## Competing interests

Conflict of Interest Statement: C.B’s department has received research grants totaling € 20,000 over the two years 2011 and 2012 from Roche Diagnostics (Mannheim, Germany). Neither C.C. nor H.P. or G.S. have received further industry sponsorship in the past five years. There was no further financing of the manuscript, no patents related to the content of the manuscript are held or have an application pending. None of the authors holds any stocks or shares in Roche Diagnostics (Mannheim, Germany). None of the authors has any non-financial competing interests to declare in relation to this manuscript.

## Authors’ contributions

CC and HP planned, conducted and wrote the study, GS performed statistical analysis and data presentation. CB participated in the development of the analytical framework for the study and contributed to the writing of the manuscript. All authors read and approved the final manuscript.

## Pre-publication history

The pre-publication history for this paper can be accessed here:

http://www.biomedcentral.com/1471-2431/13/164/prepub
